# Sporadic Cholera in London and Paris

**Published:** 1846-10

**Authors:** 


*Sporadic Cholera in London and Paris.*—It appears from the late journals that sporadic cholera has prevailed during the past summer to a very great extent in London and Paris. According to the *Gazette Medicale* (August 15th) two forms of Cholera have occurred in Paris—one characterized by great mildness and the other by great severity in the symptoms. In all instances diarrhœa had for several days preceded the attack. No case has, it is said, proved fatal.—*Med. News.*

